# Osteocalcin Mediates Biomineralization during Osteogenic Maturation in Human Mesenchymal Stromal Cells

**DOI:** 10.3390/ijms18010159

**Published:** 2017-01-17

**Authors:** Yu-Tzu Tsao, Yi-Jeng Huang, Hao-Hsiang Wu, Yu-An Liu, Yi-Shiuan Liu, Oscar K. Lee

**Affiliations:** 1 Institute of Clinical Medicine, National Yang-Ming University, Taipei 11221, Taiwan; tsaoyutzu@gmail.com; 2 Division of Nephrology, Department of Medicine, Taoyuan General Hospital, Ministry of Health and Welfare, Taoyuan 33004, Taiwan; 3 Institute of Biophotonics, National Yang-Ming University, Taipei 11221, Taiwan; hieiinuyasha@gmail.com (Y.-J.H.); haohsiang.wu@gmail.com (H.-H.W.); 4 Stem Cell Research Center, National Yang-Ming University, Taipei 11221, Taiwan; yuan_01@hotmail.com; 5 Taipei City Hospital, Taipei 10341, Taiwan; 6 Department of Medical Research, Taipei Veterans General Hospital, Taipei 11217, Taiwan; 7 Department of Orthopaedics and Traumatology, Taipei Veterans General Hospital, Taipei 11217, Taiwan

**Keywords:** mesenchymal stromal cells (MSCs), osteocalcin, osteogenic differentiation, non-collagenous protein, mineralization, hydroxyapatite, Raman spectroscopy

## Abstract

There is a growing interest in cell therapies using mesenchymal stromal cells (MSCs) for repairing bone defects. MSCs have the ability to differentiate into osteoprogenitors and osteoblasts as well as to form calcified bone matrix. However, the molecular mechanisms governing mineralization during osteogenic differentiation remain unclear. Non-collagenous proteins in the extracellular matrix are believed to control different aspects of the mineralization. Since osteocalcin is the most abundant non-collagenous bone matrix protein, the purpose of this study is to investigate the roles of osteocalcin in mineral species production during osteogenesis of MSCs. Using Raman spectroscopy, we found that the maturation of mineral species was affected by osteocalcin expression level. After osteocalcin was knocked down, the mineral species maturation was delayed and total hydroxyapatite was lower than the control group. In addition, the expression of osteogenic marker genes, including *RUNX2*, alkaline phosphatase, type I collagen, and osteonectin, was downregulated during osteogenic differentiation compared to the control group; whereas gene expression of osterix was upregulated after the knockdown. Together, osteocalcin plays an essential role for the maturation of mineral species and modulates osteogenic differentiation of MSCs. The results offer new insights into the enhancement of new bone formation, such as for the treatments of osteoporosis and fracture healing.

## 1. Introduction

Mesenchymal stromal/stem cells (MSCs) are multipotent stem cells with a high capacity for self-renewal. These cells could be isolated from human tissues like bone marrow [1,2], adipose tissue [3], umbilical cord matrix [4], and the periosteum [5]. MSCs can differentiate into adipocytes, osteoblasts, myocytes, and chondrocytes in vivo and in vitro [5,6]. Furthermore, when specific culture conditions and proper stimuli are applied in vitro, MSCs can differentiate into the cells of non-mesenchymal origin like hepatocytes and neuron cells [5]. These cells have the potential for cell therapy [7] and possess advantages in clinical applications, such as less ethical issues compared to embryonic stem cells and lack of teratoma-forming ability when transplanted in vivo [8]. In addition, various lines of study have indicated that MSCs play a crucial role in bone formation and remodeling [9].

Bio-mineralization is a process by which minerals are deposited within or outside the cells in a variety of organisms. In bone, mineralization starts from a heterogeneous solution containing calcium and phosphate ions. Hydroxyapatite (HAP), Ca_10_(PO_4_)_6_(OH)_2_, is a nature mineral form of calcium apatite and the major mineral component of bone tissue. It is located between extracellular matrixes of collagen fibers as well as embedded in non-collagenous proteins. Previous studies have shown that hydroxyapatite distribution increased with maturation of bone tissue and it can be used as biomarker during osteogenic differentiation of MSCs. Furthermore, not only hydroxyapatite but also other mineral species such as amorphous calcium phosphate (ACP), octacalcium phosphate (OCP), β-tricalcium phosphate (β-TCP), and dicalcium phosphate dehydrate (DCPD) can be found in mineral deposits [10,11]. Raman spectroscopy has been used to detect these mineral species during the process of mineralization in bone [12]. We have also developed a protocol using Raman spectroscopy to evaluate the maturation of MSCs-derived osteoblasts by monitoring the production of different mineral matrices, including hydroxyapatite, octacalcium phosphate, and β-tricalcium phosphate. Raman spectra indicated that these mineral matrices are produced at different stages of osteogenic differentiation [13]. However, the mechanism of different mineral productions is still unclear.

Non-collagenous proteins, including osteocalcin (OCN) and osteonectin (ON), represent only one-tenth of the mineralized extracellular matrix. Yet, their roles in the regulation of bone turnover and mineralization are essential [14,15]. Osteocalcin, known as bone γ-carboxyglutamic acid-containing protein (BGLAP) preferentially expressed by osteoblasts, is the most abundant non-collagenous bone matrix protein [16]. Therefore, osteocalcin is often used as a late marker for bone formation [17]. γ-Carboxyglutamic acid residues of osteocalcin are involved in calcium and hydroxyapatite binding, allowing osteocalcin deposition in the mineralized matrix. Several lines of research indicate that osteocalcin enhances bone formation. It was reported that osteocalcin improved the adherence of osteoblast-like cells on biocement [18]. Besides, osteocalcin enhances the appearance of active osteoblasts and bone healing around hydroxyapatite/collagen composites [19]. Interestingly, loss-of-function experiments in mice demonstrated that the absence of osteocalcin did not affect bone resorption but enhanced bone formation [20]. The exact role of osteocalcin in bone is not completely understood, despite its confirmed role as a regulator in bone mineralization and bone turnover [21].

The advantage of Raman spectroscopy is that it is a label-free, non-invasive measurement. This high sensitivity technique uses the inelastic scattering of light to acquire a vibrational spectrum that represents the chemical constituents of the sample. Because it can be performed with long wavelength light that has low phototoxicity, Raman spectroscopy is well suited for live cell analysis [22,23,24,25] and has been widely used as an in situ single cell detector for a variety of biological applications, such as detections of cell cycle, cell death, and identification of cell component [26,27,28]. Differentiation of MSCs into various tissue cells also possesses unique Raman signals that are associated with differentiation commitment [2,29].

Elucidation of the mechanisms underlying mineralization is crucial not only for understanding normal physiological development, but also for seeking effective treatments of impaired mineralization. Osteocalcin, the hydroxyapatite binding partner, may play an important role in the mineralization process of MSCs during osteogenic differentiation. We hypothesize that osteocalcin mediates hydroxyapatite formation during osteogenic maturation in MSCs and the aim of this study is to investigate the role of osteocalcin in mineralization and osteogenic maturation of MSCs.

## 2. Results

### 2.1. In Vitro Analysis of MSCs under Osteogenic Induction on Quartz Coverslips

**Figure 1:**
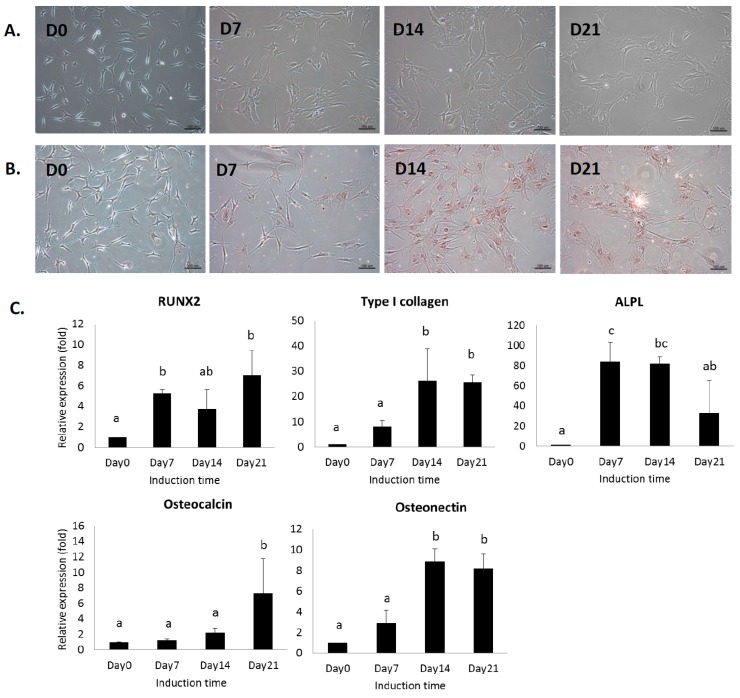
(**A**) Photomicrograph of mesenchymal stromal cells (MSCs) cultured on quartz coverslips in osteogenic induction medium at day 0, 7, 14, and 21. The halo regions surrounding cell edges of MSCs are the artifacts due to phase contrast microscope; (**B**) alizarin red S staining of MSCs during osteogenic differentiation. Scale bar: 100 μm; (**C**) gene expression of osteoblast-related markers on quartz coverslips. Data are shown as mean ± SD (*n* = 3). One-way analysis of variance (ANOVA) followed by Tukey’s post-hoc tests was performed for multiple comparisons. Different letters represent significant differences between groups (*p* < 0.05). For example, a and b represent significant difference, a and bc represent significant difference, whereas a and ab do not.

Conventional biochemical analysis was used to assess the osteogenic differentiation of human MSCs cultured on quartz coverslips. The morphology of MSCs changed from spindle-like to flattened osteoblast-like morphology during osteogenic differentiation on quartz coverslips (Figure 1A). Alizarin red S-staining was used to confirm the presence of calcific deposition from the cells of osteogenic lineage (Figure 1B). Gene expression of osteoblast-related markers was analyzed by real-time PCR. The gene expression of *RUNX2*, alkaline phosphatase (*ALPL*), type I collagen alpha 1 (*Col1A1*), osteonectin (*ON*), and osteocalcin (*OCN*) was upregulated after the induction (Figure 1C). These results suggested the progression of MSCs into a more differentiated stage as mature osteoblasts.

### 2.2. Gene Expression Analysis of MSCs after Osteocalcin Knockdown

**Figure 2:**
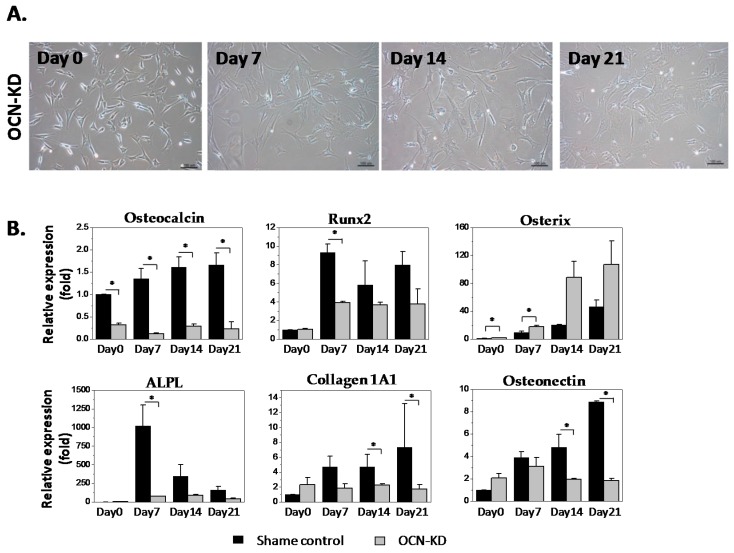
(**A**) Photomicrograph of osteocalcin-knockdown (OCN-KD) cultured on quartz coverslips in osteogenic induction medium at day 0, 7, 14, and 21. Scale bar: 100 μm. The halo regions surrounding cell edges of MSCs are the artifacts due to phase contrast microscope; (**B**) the gene expression of Sham and OCN-KD groups. Data are shown as mean ± SD (*n* = 3). Student’s *t*-test was used for two group analysis with significant difference indicated by asterisks (* *p* < 0.05).

To study the role of osteocalcin in osteogenic differentiation and mineralization of MSCs, human MSCs were treated with siRNA to knockdown the gene expression of osteocalcin. Cell morphologies of sham control group and osteocalcin-knockdown (OCN-KD) group undergoing osteogenic differentiation for three weeks on quartz coverslips are shown in Figure 2A. Gene expression of osteocalcin after siRNA treatment was about 25% of that of sham control group and the treatment of siRNA can stay effective for three weeks after osteogenic induction (Figure 2B). Expression of osteoblast-related genes in OCN-KD, including *RUNX2*, type I collagen alpha 1, alkaline phosphatase, and osteonectin was downregulated compared to the sham control. On the other hand, gene expression of osterix was upregulated after the knockdown. Nevertheless, gene expression of those osteogenic markers remained the tendency to increase along the differentiation course in both OCN-KD and the control groups. In short, MSCs with low expression of osteocalcin still possessed the potential toward osteogenic differentiation; several osteogenic marker genes were even downregulated after osteocalcin was knocked down.

### 2.3. Background Signals of Raman

**Figure 3:**
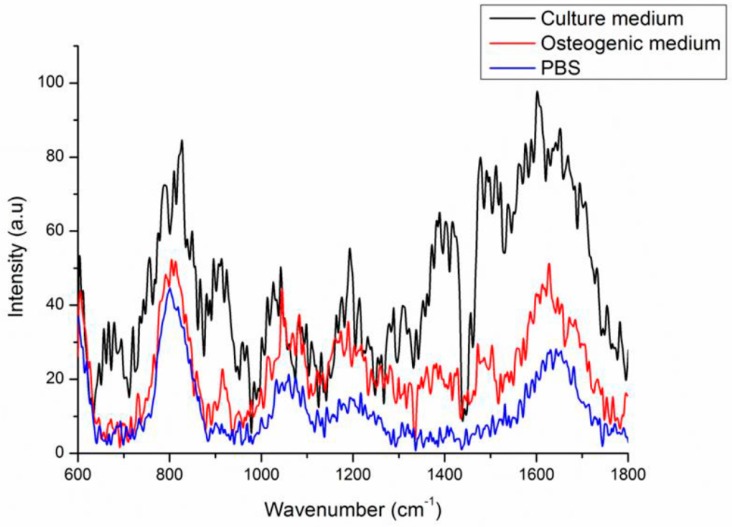
Raman spectra of background signals of quartz coverslips with culture medium, osteogenic induction medium, and PBS.

In order to effectively subtract Raman background signal from the signals of mineral species, we investigated the Raman spectrum background signal of quartz coverslips with culture medium containing serum, quartz coverslips with osteogenic medium, and quartz coverslips with phosphate buffered saline (PBS) (Figure 3). The Raman spectra of quartz coverslips had two apparent peaks at 800 and 1050 cm^−^^1^ which were the quartz material signals. For quartz coverslips with PBS and osteogenic medium groups, the background signals were quite similar; while the culture medium group had a pattern of background signal distinct from others due to the presence of serum. We chose quartz coverslips with osteogenic medium to be the background control instead of quartz coverslips with PBS due to that MSCs could live longer in osteogenic medium than in PBS during the process of Raman spectra acquisition. In the range of 900–1020 cm^−^^1^, which was reported as the range of the mineral species signals, the Raman background signal was smooth without noise; therefore, this range is suitable for investigating the changes of mineral species during osteogenic differentiation of MSCs.

### 2.4. Raman Spectra of MSCs with Osteocalcin Knockdown

**Figure 4:**
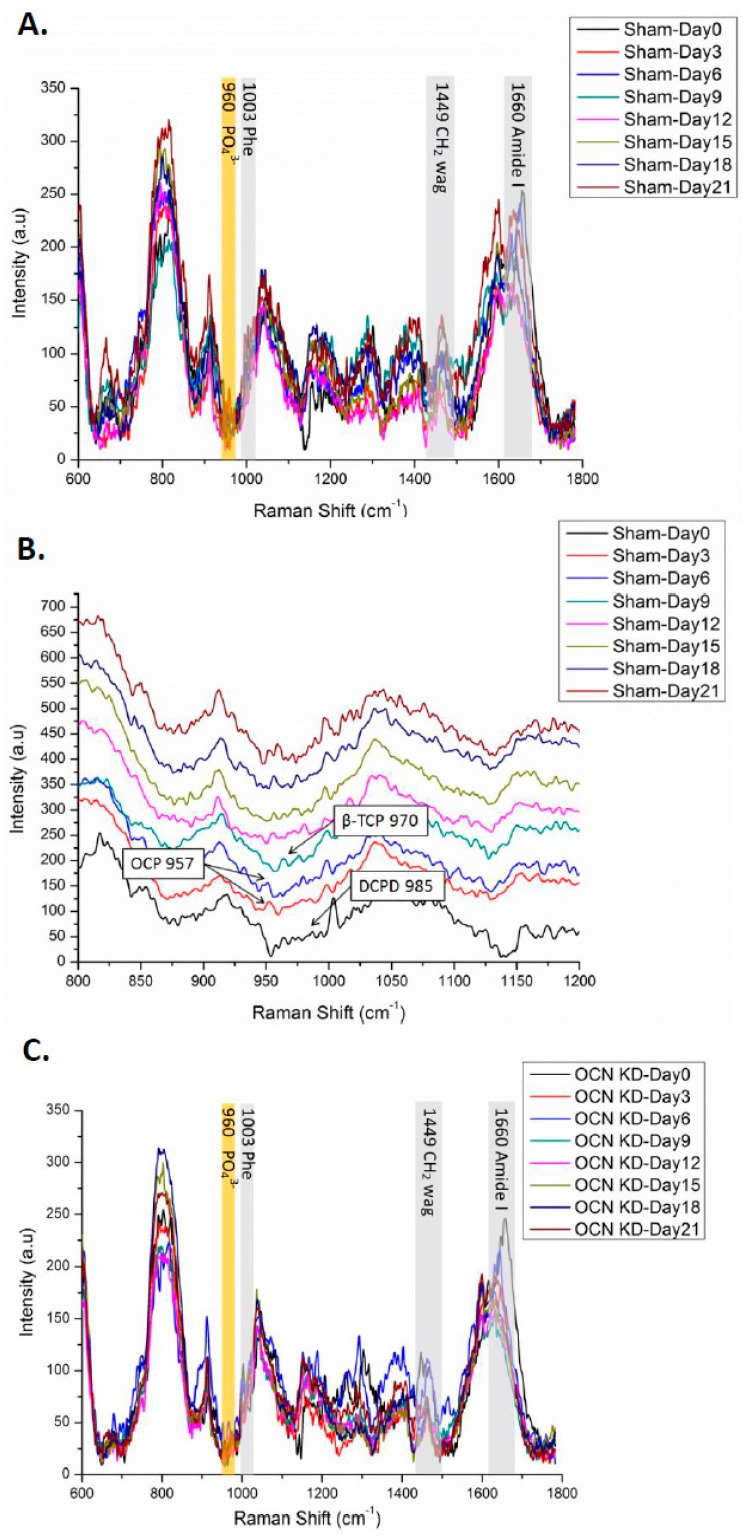

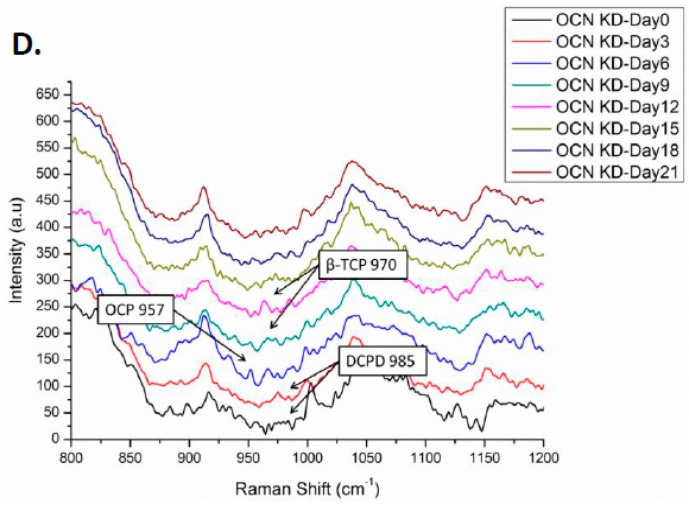
(**A**,**B**) Raman spectra of the sham control group during osteogenic differentiation: (**A**) complete view from 600 to 1800 cm^−1^; (**B**) the detail region from 800 to 1200 cm^−1^ in stack diagram; (**C**,**D**) Raman spectra of the OCN-KD group; (**C**) complete view from 600 to 1800 cm^−1^; (**D**) the detail region from 800 to 1200 cm^−1^ in stack diagram.

When MSCs were cultured on quartz coverslips, Raman signals of cellular components including proteins like phenylalanine (Phe) at 1003 cm^−^^1^, carbohydrates like CH_2_ wag at 1449 cm^−^^1^, and amide I at 1660 cm^−^^1^ could help us to assess the maturation of osteogenic differentiation (Figure 4A,C). In order to elucidate the relationship between osteocalcin and mineralization during osteogenic differentiation of MSCs, Raman spectra of both sham and OCN-KD groups were collected every three days until 21 days after the induction. Raman spectra obtained from at least 10 locations on the surface of differentiating cells were averaged. All data were evaluated by routine signal processing including smoothing, cosmic ray removal, and multipoint baseline correction. The regions of Raman spectra are shown in gray for cellular components and in yellow for mineral species.

To investigate the role of osteocalcin in the mineralization process of osteogenic differentiation, the region from 800 to 1200 cm^−^^1^ of Raman spectrum was dissected in detail as depicted in Figure 4B,D. In the sham group, the peak at 985 cm^−^^1^ representing dicalcium phosphate dehydrate was observed in undifferentiated MSCs. Three days after the induction, octacalcium phosphate signal peaked at 957 cm^−^^1^ emerged until day six. Then β-tricalcium phosphate at 970 cm^−^^1^ was identified at day nine. The intensity of hydroxyapatite signal at 960 cm^−^^1^ continually increased as a function of induction time during osteogenic differentiation. However, we could not identify amorphous calcium phosphate signal at 952 cm^−^^1^ in Raman spectra during osteogenic differentiation of MSCs [30]. In OCN-KD group, we found that the formation of mineral species was delayed. Dicalcium phosphate dehydrate got an obvious peak until day three after the induction. The octacalcium phosphate signal was postponed until day six; the β-tricalcium phosphate emerged of a longer period, until day 12; and the intensity of hydroxyapatite increased from day 15.

**Figure 5:**
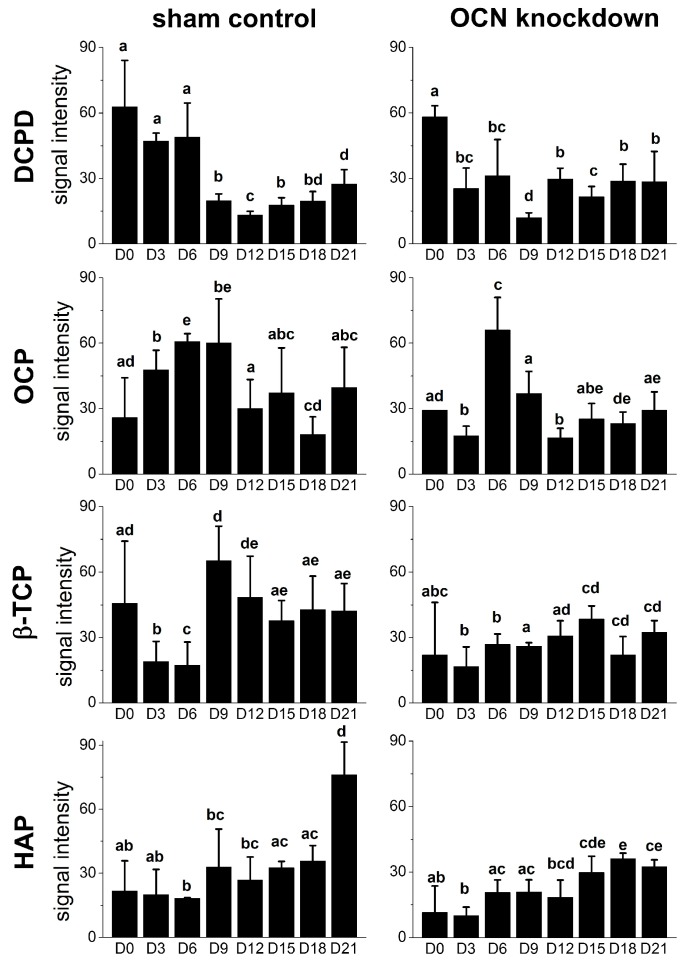
The intensities of peaks of mineral species signals dicalcium phosphate dehydrate (DCPD) at 985 cm^−1^, octacalcium phosphate (OCP) at 957 cm^−1^, β-tricalcium phosphate (β-TCP) at 970 cm^−1^, and hydroxyapatite (HAP) at 960 cm^−1^ along the time course of osteogenic differentiation. Spectra were collected on day 0, 3, 6, 9, 12, 15, 18, and 21 after the induction. Data are shown as mean ± SD. One-way analysis of variance (ANOVA) followed by running Tukey’s post-hoc tests was performed for multiple comparisons. Different letters represent significant differences between groups. For example, a and b represent significant difference, a and bc represent significant difference, whereas ab and bcd do not. A *p* value less than 0.05 is defined as statistically significant; D = Day.

**Figure 6:**
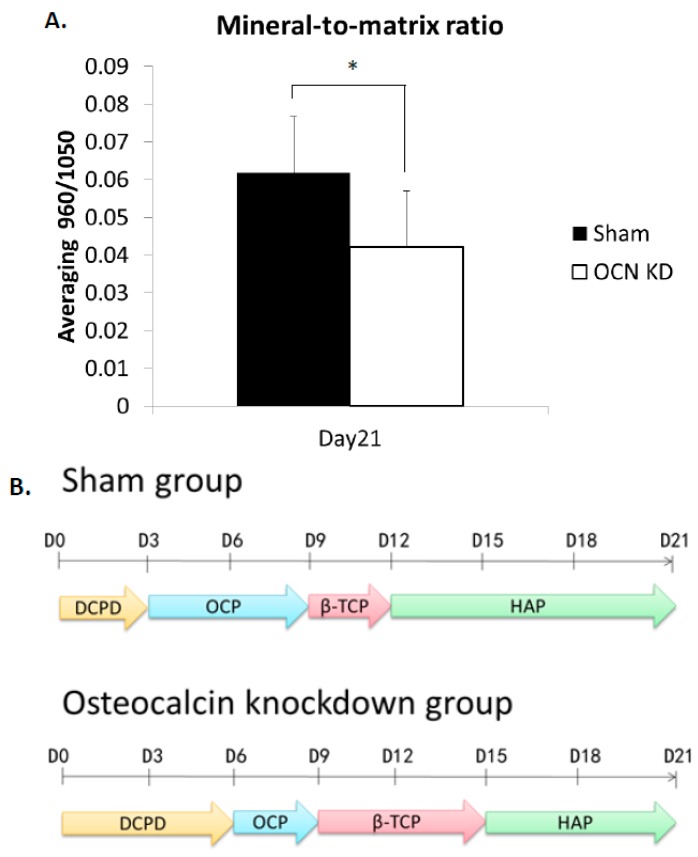
(**A**) The mineral-to-matrix ratios obtained from Raman intensity ratio of HAP/quartz (960/1050). Data are shown as mean ± SD (*n* = 3) (* *p* < 0.05); (**B**) the mineral species maturation process in MSCs of sham and OCN-KD during osteogenic differentiation; D = Day.

In order to compare Raman signals of mineral species, the mineral-to-matrix ratio of hydroxyapatite/quartz was calculated as the relative intensity of signal ratio 960/1050 (cm^−^^1^/cm^−^^1^). The intensity ratio of OCN-KD group was significantly lower than that of sham group (Figure 5 and Figure 6A). The time points of mineral species maturation were summarized in Figure 6B. The sequence of mineral species maturation was dicalcium phosphate dehydrate (DCPD), octacalcium phosphate (OCP), β-tricalcium phosphate (β-TCP), and finally hydroxyapatite. The Ca^2+^/PO_4_ ratio was higher and higher as consequence. When osteocalcin was insufficiently expressed, the maturation of mineralization was delayed and hydroxyapatite production was lower compared to the sham control.

## 3. Discussion

Raman spectroscopy is a label-free, non-disruptive measurement and has been proven to be suited for live cell analysis without phototoxicity. Raman spectroscopy can be used to characterize the differentiation process of stem cells over the period of 14 days [31,32]. Considering cell viability and the background noise of Raman signal, osteogenic induction medium is the most proper medium for Raman measurement of MSCs in the present study. Matrix band is used to normalize the Raman spectrum by integrating the area of intensity graph. We normalized all spectra by the area of the 1050 cm^−1^ band, which is the signal of quartz coverslips. The intensity of the peak is stable therefore the normalization can provide robust data with respect to the matrix content. Amide I band from 1500 to 1800 cm^−1^ is also a matrix content and commonly used for normalization. Since amide I is related to collagen protein, we did not choose the amide I signal for normalization in the present study. Phe is also considered as matrix content, but it could not be relied upon because the signal could be contributed from non-collagenous proteins. CH2 wag at 1449 cm^−1^ is another measured matrix used in many studies, but in our current experimental setup, the signal did not show up constantly during the course of differentiation. For the above reasons, we suggest that the normalization could be properly adjusted based on the peak at 1050 cm^−1^, the background signal from quartz coverslips.

The results showed that dicalcium phosphate dehydrate is present in undifferentiated MSCs, then octacalcium phosphate emerges at the early stage of osteogenic differentiation, followed by β-tricalcium phosphate for a short period, and afterward hydroxyapatite keeps increasing at the late stage of the differentiation. Another candidate for hydroxyapatite precursor, negligible production of amorphous calcium phosphate during osteogenesis of MSCs, implies a content-dependent phenotype by Raman spectroscopy. It is possible that either the amount of amorphous calcium phosphate is too low to be detected in differentiating MSCs or there are some intrinsic differences between osteogenic differentiations in vivo and in vitro. To further analyze the trace of mineral species, we will combine energy-dispersive X-ray spectroscopy and scanning electron microscopy (EDS-SEM) with Raman spectroscopy in future studies.

During the crystal growing of bio-mineralization process, osteocalcin has been reported to accelerate nucleation of hydroxyapatite and inhibit hydroxyapatite precipitation. In the OCN-KD MSCs group studied in the present manuscript, the maturation of mineral species was postponed and the mineral-to-matrix ratio was significantly lower compared to the control group (Figure 5 and Figure 6). These phenomena confirm that osteocalcin is important in regulating mineralization process during the osteogenic differentiation of MSCs. Bone has been recently recognized as an endocrine organ. As an endocrine hormone, osteocalcin is reported to mediate bone and glucose homeostasis. For example, insulin secreted from pancreas activates insulin receptors in osteoblasts and therefore inhibits Twist2 expression. Since Twist2 is an inhibitor of Runx2, Runx2 is upregulated and consequently osteocalcin expression increases. Acting as a positive feedforward loop, upregulated osteocalcin is decarboxylated by osteoclasts from bone matrix and released into circulation to further stimulate insulin secreted from pancreas [33]. In this study, we found that osteocalcin knockdown downregulated *RUNX2*, therefore other osteoblast-related genes were also downregulated. The results indicate that this signaling loop is mediated via other mechanisms in vitro, instead of through the insulin secretion from pancreas. Interestingly, upregulation of osterix after osteocalcin knockdown as well as the enhancement of bone formation in the absence of osteocalcin in vivo [20] indicate a possible biphasic regulation in bone formation that osteocalcin may be involved. Since bio-mineralization is a highly regulated process that occurs through the interaction of negatively charged functional groups of organic macromolecules with phosphate and calcium, the underlying molecular mechanism of bio-mineralization that osteocalcin mediates, together with the effect of calcium and phosphate, also warrants further investigation.

Previous study in our laboratory demonstrated that Raman spectroscopy has higher sensitivity at the single-cell level and the mineralization level of differentiating MSCs is easier to quantitatively evaluate by Raman spectroscopy compared with the conventional methods [13]. Moreover, owing to the newly modified setup of Raman spectroscopy, we identified the peak of mineral species dicalcium phosphate dehydrate (DCPD) that we cannot detect before. Furthermore, we demonstrate that osteocalcin is important in the mineralization process. The finding could help us in further understanding the mechanism of mineralization as well as for the continued development of new markers for bone turn over to increase the knowledge of metabolic bone diseases like osteoporosis.

## 4. Materials and Methods

### 4.1. Culture Maintenance and Osteogenic Differentiation of MSCs

Commercially available human MSCs (Steminent Biotherapeutics Inc., Taipei, Taiwan) were maintained in Iscove’s Modified Dulbecco’s medium (IMDM; Gibco, Grand Island, NY, USA) supplemented with 10% ES fetal bovine serum (ES-FBS; Sigma-Aldrich, St. Louis, MO, USA), 10 ng/mL basic fibroblast growth factor (bFGF; R&D Systems, Inc., Minneapolis, MN, USA), and 100 U penicillin, 1000 U streptomycin, 2 mM l-glutamine (PSG; Gibco).

Induction into mesodermal lineage progenies was carried out according to our previous protocols [4]. To induce osteogenic differentiation, MSCs were cultured to the density of approximately 50% confluence on quartz coverslips before the treatment with osteogenic induction medium which consists of IMDM supplemented with 0.1 μM dexamethasone (Sigma-Aldrich), 0.2 mM ascorbic acid (Sigma-Aldrich), and 10 mM β-glycerol phosphate (Sigma-Aldrich). MSCs were treated with osteogenic induction medium for three weeks and the medium was changed every three days.

### 4.2. siRNA Transfection

One day prior to the transfection, the expansion medium of MSCs was changed to antibiotic-free growth medium (IMDM with 2% ES-FBS and 10 ng/mL bFGF). siRNA targeting BGLAP was transfected into MSCs according to the manufacturer's instructions. Briefly, 50 nM of siRNA against OCN (GACCCAGGCGCUACCUGUAUCAAUG and CAUUGAUACAGGUAGCGCCUGGGUC, HSS184525, Stealth RNAi^TM^, Thermo Fisher Scientific, Waltham, MA, USA) was transfected using Lipofectamine^®^ RNAiMAX Transfection Reagent (Invitrogen, Carlsbad, CA, USA) into MSCs at 60% confluence for 16 h at 37 °C. Cells were then washed with PBS and cultured in osteogenic induction medium afterward. Medium GC scramble was used as sham control (20 μM, Invitrogen). Expression of osteocalcin was analyzed every 7 days after the induction to confirm the consistency of knockdown efficiency along the time course of the differentiation.

### 4.3. RNA Extraction and Real-Time Quantitative Polymerase Chain Reaction

Total RNA was extracted using the RNeasy^®^ Mini kit (QIAGEN, Valencia, CA, USA). RNA sample were reverse-transcribed to complementary DNA (cDNA) using reagents (Genemark Technology, Taipei, Taiwan) according to the manufacturer instructions. Real-time quantitative polymerase chain reaction was performed on ABI Step One Plus Real Time PCR System. The whole reaction was amplified at 95 °C for 20 s, followed by 40 cycles of 1 s at 95 °C and 20 s at 60 °C. The average threshold cycle (*C*_t_) for each gene was normalized by Glyceraldehyde 3-phosphate dehydrogenase (*GAPDH*) as housekeeping gene for analysis. The primer sequences and corresponding probes were listed in Table A1.

### 4.4. Alizarin Red S Staining

For evaluation of mineralization, cells cultured on quartz coverslips were rinsed twice with PBS, fixed with 10% formaldehyde for 20 min, and washed twice with PBS. Mineralization matrix was analyzed by using 40 mM Alizarin red S solution (Sigma-Aldrich) for 20 min at room temperature. Cells were washed by distilled water carefully for four times and then water was completely removed for further analysis.

### 4.5. Raman Spectroscopy

Jobin Yvon LabRam HR 800, HORIBA was used as the setup of Raman spectroscopy. An 18-mW He-Ne laser operating at 632.8 nm was used to provide the Raman excitation light source for biological samples. With the rotatable λ/2-plate, the polarization direction of the incident beam could be adjusted continuously. The objective lens we used was 60× water immersion M-Plan objective (NA = 0.9). With the analyzer in the back scattered path, the polarization direction of the backscattered signal can be set. Raman signals were collected from the spectrum between 600 and 1800 cm^−^^1^ with a resolution of approximately 1 cm^−^^1^ and the integration time of 75 s.

For sample preparation, MSCs seeded on 2 cm × 2 cm quartz coverslips were under osteogenic induction for 3 weeks and Raman spectra were collected every three days. Before analysis, cells were washed twice with fresh osteogenic induction medium and the coverslips was placed on the quartz slide. We added osteogenic induction medium on the coverslips to avoid cell death during the acquisition. Raman spectra were obtained from at least 10 locations selected from the surface of cell samples for each sample preparation. Three independent sample preparations were performed. All data were speculated by routine signal processing, including smoothing, cosmic ray removal, and multipoint baseline correction by Labspec 5.0 (HORIBA, Kyoto, Japan). The spectra were normalized by the area of 1050 cm^−^^1^ band.

## 5. Conclusions

Raman spectra of mineral species produced by differentiating MSCs of control and osteocalcin knockdown groups during osteogenic differentiation were analyzed. The peaks of mineral species signals were dicalcium phosphate dehydrate (DCPD) at 985 cm^−^^1^, octacalcium phosphate (OCP) at 957 cm^−^^1^, β-tricalcium phosphate (β-TCP) at 970 cm^−^^1^, and hydroxyapatite (HAP) at 960 cm^−^^1^; these signals emerged sequentially along the time course of osteogenic differentiation. When osteocalcin gene expression level was low, the time points that mineral species signals appeared were delayed compared to the control group. Moreover, the mineral-to-matrix ratio was lower than the control group after three-week osteogenic induction. In addition, knockdown of osteocalcin downregulated gene expression of osteogenic markers, including *RUNX2*, alkaline phosphatases, osteonectin, and type I collagen, but upregulated that of osterix. Our results confirm the influence of osteocalcin on hydroxyapatite formation by demonstrating that osteocalcin modulates the mineral species maturation during osteogenic differentiation of MSCs. Moreover, we supplement the evidence on the molecular basis that osteocalcin not only is one of the late protein products of osteogenesis but also a signal to modulate the expression of transcription factors for osteogenic differentiation. The study provides further understandings of bio-mineralization process that osteocalcin involved.

## Abbreviations

MSCs	mesenchymal stromal cells
OCN/BGLAP	osteocalcin/bone γ-carboxyglutamic acid-containing protein
HAP	hydroxyapatite
ACP	amorphous calcium phosphate
OCP	octacalcium phosphate
β-TCP	β-tricalcium phosphate
DCPD	dicalcium phosphate dehydrate
ON	osteonectin
ALPL	alkaline phosphatases
COL1A1	type I collagen alpha 1
OCN-KD	osteocalcin knockdown
PBS	phosphate-buffered saline
FBS	fetal bovine serum

## Appendix A

**Table A1:** Primer sequences and probes used for real-time PCR analysis.

Gene	Oligonucleotide Sequence	Probe
*GAPDH*	5′-AGCCACATCGCTCAGACAC-3′/5′-GCCCAATACGACCAAATCC-3′	60
*RUNX2*	5′-CACCATGTCAGCAAAACTTCTT-3′/5′-TCACGTCGCTCATTTTGC-3′	41
Osteocalcin	5′-GGCGCTACCTGTATCAATGG-3′/5′-TCAGCCAACTCGTCACAGTC-3′	1
Osteonectin	5′-GTGCAGAGGAAACCGAAGAG-3′/5′-TGTTTGCAGTGGTGGTTCTG-3′	77
*ALPL*	5′-AGAACCCCAAGGCTTCTTC-3′/5′-CTTGGCTTTTCCTTCATGGT-3′	31
Type I collagen alpha 1	5′-CCCCTGGAAAGAATGGAGAT-3′/5′-AATCCTCGAGCACCCTGAG-3′	60
Osterix/*SP7*	5′-GACTGCAGAGCAGGTTCCTC-3′/5′-TAACCTGATGGGGTCATGGT-3′	43
